# Extent of Digital Health Fragmentation and Potential Implications for Antimicrobial Prescribing: Rapid Evidence Review

**DOI:** 10.2196/94014

**Published:** 2026-10-06

**Authors:** Verena Schneider, Akish Luintel, Bethan Balch, Shivani Gangadia, Grainne Brady, Emma McGuire, Gwenan M Knight, Laura Shallcross, Steve Harris, Cecilia Vindrola-Padros

**Affiliations:** 1 Department of Targeted Intervention University College London London, England United Kingdom; 2 University College London Hospitals NHS Foundation Trust London, England United Kingdom; 3 Division of Psychiatry University College London London, England United Kingdom; 4 London School of Hygiene & Tropical Medicine London, England United Kingdom; 5 Institute of Health Informatics University College London London, England United Kingdom

**Keywords:** electronic health records, interoperability, data sharing, systems integration, medical record linkage, United Kingdom, England, antimicrobial prescribing, antimicrobial stewardship, patient safety

## Abstract

**Background:**

Prior microbiology results, resistance patterns, and antimicrobial exposure are central to safe and effective antimicrobial prescribing. Digital health fragmentation refers to the dispersal of patient data across multiple electronic systems and the associated challenge of accessing complete information at the point of care. Antimicrobial prescribing for infections represents a critical use case to investigate the impact of digital health fragmentation on patient care. While interoperability has been studied in the context of patient safety, no review has described digital health fragmentation within the United Kingdom and examined its impact on antimicrobial prescribing and antimicrobial stewardship (AMS).

**Objective:**

This study aimed to (1) characterize the extent of digital health fragmentation in the United Kingdom, (2) summarize the available evidence on its impact on AMS and prescribing practices in high-income countries, and (3) identify potential solutions.

**Methods:**

A rapid review of the peer-reviewed literature was conducted following published guidance for rapid reviews and the PRISMA (Preferred Reporting Items of Systematic Reviews and Meta-Analyses) statement. MEDLINE ALL and PsycInfo were searched on August 19, 2025, using search terms relating to digital health fragmentation or interoperability, patient safety, and antimicrobial use. Searches were limited to English-language publications from 2015 (for characterizing the recent trends or current state of digital health fragmentation in the United Kingdom) or 2010 onward (for AMS-related impacts and solutions). Screening was conducted by 4 researchers following predefined inclusion and exclusion criteria. Extracted data were synthesized narratively through framework analysis. Study quality was appraised using the Mixed Methods Appraisal Tool.

**Results:**

Fourteen studies met the inclusion criteria. Ten studies described the extent and nature of digital health fragmentation in the United Kingdom. Digital health fragmentation affects a large number of patients and is linked to clinical care efficiency, quality, and safety risks, including limited access to external clinical records, missing or incomplete information, duplicate investigations, delays in decision‑making, and substantial time spent searching for data. Evidence specific to antimicrobial prescribing was limited (4 studies) but indicated that AMS relies on information spread across multiple systems, with poor interoperability disrupting workflows, hindering communication, and undermining stewardship activities. Only 1 study reported the development of a digital tool designed to address digital health fragmentation and support AMS.

**Conclusions:**

Digital health fragmentation negatively affects patient care across the United Kingdom, yet evidence on how it impacts AMS remains scarce. Given the urgency of the global antimicrobial resistance crisis, future research should therefore quantify the scale and impact of digital health fragmentation for AMS to inform investment and innovation in digital infrastructure and clinical-supportive solutions.

**Trial Registration:**

PROSPERO CRD420251126067; https://www.crd.york.ac.uk/PROSPERO/view/CRD420251126067

## Introduction

A substantial number of patients in the United Kingdom are being cared for by multiple health care providers (ie, National Health Service [NHS] trusts). For example, in 2017/2018, a quarter of patients received care from more than 1 trust, with 9.1% of clinical encounters spanning different electronic health record (EHR) systems [1]. This can result in “digital health fragmentation” (Table 1 defines terms used in this paper), where patient data are dispersed across multiple digital systems with limited interoperability, data availability, and/or usability. Consequently, clinical information can be incomplete and difficult to access or use within clinical workflows, ultimately hindering patient care. This has particular implications for safe antimicrobial prescribing, where rapid access to comprehensive patient information is critical. Timely and appropriate selection of antibiotic therapy is critical in suspected infection and can be lifesaving [2]. Conversely, inappropriate antibiotic choices can lead to treatment failure, preventable adverse drug events, and the emergence and spread of antimicrobial resistance (AMR) [3]. Safe prescribing, therefore, depends on timely access to patients’ complete clinical histories, including prior microbiology results and antibiotic use [4]. Without these data, clinicians cannot reliably assess the risk of resistance or select the most effective therapy. This may also lead to default use of broader-spectrum antibiotics, inadvertently undermining stewardship goals, including the 70% global 2030 target for Access antibiotics [5]. In the context of a global AMR crisis, and rising resistance rates threatening the effectiveness of routine medical care [6,7], the consequences of incomplete information can be particularly serious, underscoring the need for reliable access to patient records across organizational boundaries.

**Table 1:** Used terms and definitions.

Term	Definition
Antimicrobial prescribing	Prescribing antibiotics or other antimicrobials in clinical practice. Appropriate antimicrobial prescribing refers to prescribing that adheres to guidelines and is clinically justified based on patient history, severity, microbiology, and local resistance patterns.
Antimicrobial stewardship	A coordinated set of activities aimed at improving appropriate antimicrobial use, optimizing therapy, reducing resistance, and minimizing harm.
Digital health fragmentation	The dispersal of patient data across multiple electronic systems alongside poor interoperability, data availability, and/or usability, resulting in incomplete, inaccessible, or unusable information for decision-making.
Electronic health record system	Digital system used to store and manage patient clinical information (eg, medications, allergies, laboratory results, and clinical notes). In the paper form, this was previously referred to as patient charts or notes.
Integration (of systems)	Technical linking of systems so that they behave as if part of the same platform.
Interfacing	A more limited technical connection between systems, often used when full integration is not possible. “A strategy that involves linking standalone systems developed separately for different purposes (usually but not always) from different suppliers so that they can exchange information. Standards that guide this information exchange can connect different systems and make them interoperable.” [8]
Interoperability	“The capability of people involved in the provision and receipt of care to interact and complete a task across software and organisational boundaries; and use equipment, systems, or products from different vendors, which operate together in a coordinated fashion, with minimal to no human intervention.” [9]
Multimodular integrated solutions	Multimodular systems “usually take the shape of different system modules (of which ePrescribing may be one) from a single vendor functioning as an integrated whole [eg, an electronic health record (EHR)] with one underlying code and database.” [8]
Vendor	The commercial supplier or developer of a digital system. Different vendors provide different electronic systems.

This review uses digital health fragmentation as a broad umbrella term that encompasses several distinct, yet related, concepts, shown in the conceptual framework in Figure 1. Patient data become spread across multiple electronic systems, for example, either because care is spread across health care providers or because a single organization or provider uses multiple internal systems. This structural condition can limit access to patients’ complete data at the point of care. The extent to which this results in digital health fragmentation depends on 2 further factors: technical fragmentation and workflow fragmentation. Technical fragmentation includes interoperability, that is, the extent to which systems can exchange data, and data availability, that is, whether the necessary data are accessible and complete at the point of care. However, interoperability and data availability are not sufficient on their own to prevent fragmentation: workflow fragmentation, that is, limited usability even in otherwise interoperable systems, can still contribute to digital health fragmentation.

**Figure 1:**
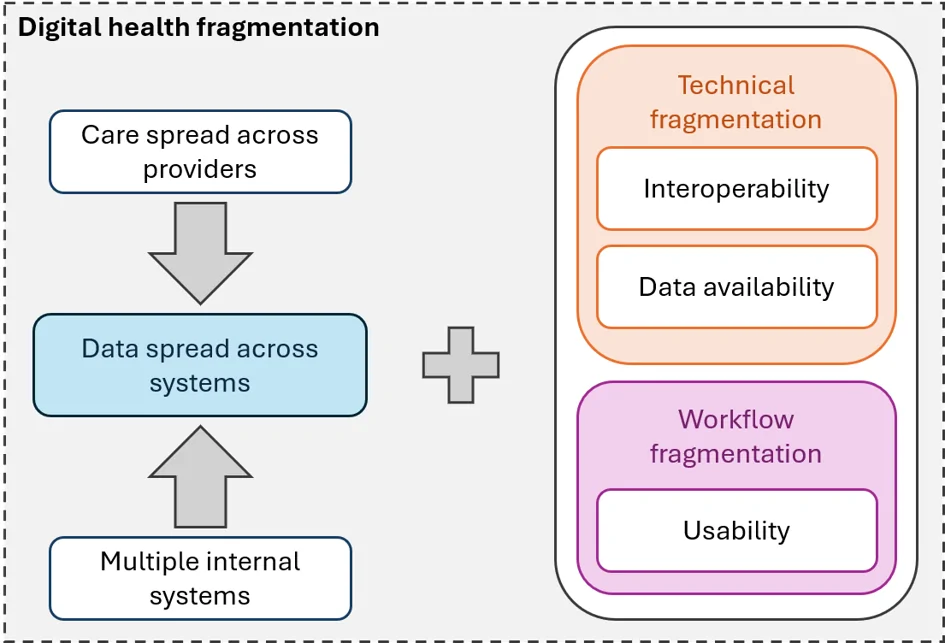
Conceptual framework of digital health fragmentation. Digital health fragmentation occurs when patient data are dispersed across multiple electronic systems alongside technical fragmentation (eg, poor interoperability and/or data availability) and/or workflow fragmentation (eg, poor usability).

A growing body of literature has examined the implications of digital health fragmentation in terms of limited interoperability. A recent systematic review found that good interoperability was associated with improvements in medication safety and data accuracy, while evidence for broader impacts on patient safety and care quality remained unclear [10]. Although these findings are likely relevant to antimicrobial prescribing and AMS, the specific relationship between digital health fragmentation and AMS has not been explored systematically, leading to gaps in our knowledge of where and what to target, as well as the size of the impact. AMS represents a valuable use case for understanding the impacts of digital health fragmentation, as infections are managed across health and social care settings (ie, as opposed to conditions that are primarily managed in one setting), requiring information to be accessible across organizational boundaries. Moreover, an exclusive focus on technical interoperability may overlook other contributors to digital health fragmentation and may limit our understanding of the specifics of the problem and how to address it. The existing evidence base is also limited by its geographical focus: most studies included in the previous review were conducted in the United States, with only 1 undertaken in the United Kingdom [10]. The impact of digital health fragmentation is likely to be context-dependent, influenced by health system organization, digital infrastructure, and patterns of care. Furthermore, additional UK evidence may have emerged since the publication of the review. Therefore, an updated overview of digital health fragmentation in the UK context, particularly since the more widespread use of EHRs, is needed.

The aim of this review was to characterize the extent and impact of digital health fragmentation within the United Kingdom and to understand its implications on antimicrobial prescribing and stewardship in high-income country health care settings. In addition, the review sought to identify any strategies or interventions that have been developed to address fragmentation in this context. Given the uncertain volume and evolving but potentially limited evidence base in this specific context, a rapid review approach was considered appropriate. Rapid reviews are recommended when the scope and volume of evidence are unclear, allowing iterative refinement of the scope, and can inform decisions about whether a full systematic review is subsequently warranted [11].

Specifically, the review aimed to answer the following research questions:

1. What is the extent (quantitative and qualitative) of digital health fragmentation in the United Kingdom, that is:
  What is the scale of care fragmentation (eg, patients accessing care across multiple NHS health care organizations) and how does this impact access to comprehensive and accurate patient data?
  What is the reported impact of digital fragmentation on care and patient safety outcomes?
2. What impact (qualitative or quantitative) does digital fragmentation have on antimicrobial prescribing and AMS in high-income countries?
3. What solutions or interventions have been explored to reduce digital fragmentation for antibiotic prescribing, and what evidence exists regarding their effectiveness in high-income countries?

## Methods

### Study Design

This rapid review was prospectively registered with the International Prospective Register of Systematic Reviews (PROSPERO; CRD420251126067). Two amendments were made to the protocol: MEDLINE was searched instead of PubMed and inclusion criteria for research question 1 were limited to studies published from 2015 onward. Reporting followed the interim guidance for rapid reviews [12] and the PRISMA (Preferred Reporting Items of Systematic Reviews and Meta-Analyses) statement [13] (see Multimedia Appendix 1 for the PRISMA checklist).

### Search Strategy

Search strategies were developed iteratively with input from the research team and piloted. MEDLINE ALL and PsycInfo were searched via Ovid on August 19, 2025, using combinations of terms relating to digital health fragmentation, interoperability, patient safety, antimicrobial prescribing, and antimicrobial stewardship (AMS) (see Multimedia Appendix 2 for full search strategy). Databases were searched for papers published from January 1, 2010, with no search end date restrictions, and an English language filter was applied. Citations and references of included papers were screened to identify any additional papers meeting the inclusion criteria. Additionally, the Cochrane Library was searched on August 19, 2025, and any relevant systematic reviews identified on any of the databases were screened for further relevant papers.

### Study Selection

#### Overview

Rayyan software (Rayyan Systems Inc) was used for manual deduplication and screening of records [14]. After removing duplicates, titles and abstracts of retrieved records were screened for eligibility individually by 2 reviewers (VS and GB), followed by full-text screening of potentially relevant papers by a single reviewer (VS), and excluded records were dual-screened by second reviewers (BB and SG) at both the title and abstract (2473/9889, 25%) and full-text review (11/42, 25%) stages. Any discrepancies and borderline papers were discussed until a consensus was reached. This approach differs from the dual-screening process recommended in the Cochrane systematic review methodology but is a common approach in rapid reviews to streamline the process while ensuring accuracy [11,15]. All included papers were double screened by second reviewers (BB and SG).

#### Eligibility Criteria

Papers were eligible for inclusion if they were peer-reviewed original research using qualitative, quantitative, or mixed methods, conducted in high-income countries and published in English. Furthermore, included research needed to address at least one of the following: (1) the state, scale, or impact of digital health fragmentation in the NHS in the United Kingdom; (2) the impact of digital health fragmentation on antimicrobial prescribing, resistance, or AMS in high-income countries; or (3) interventions or strategies designed to reduce digital health fragmentation in relation to AMS in high-income countries.

An inclusive approach was adopted. Studies were eligible even when digital health fragmentation was not the primary focus, provided that it was identified as a contributing factor to patient safety or AMS-related outcomes. However, the strength of such indirect evidence was considered limited and noted during quality appraisal.

Excluded were alternative publication types (eg, gray literature, book chapters, conference abstracts, editorials, commentaries, and opinion pieces), secondary research (eg, reviews and meta-analyses), and studies focused solely on human communication failures rather than system-level digital health fragmentation. Studies examining interoperability between a single innovative system and a local EHR only (eg, stand-alone decision support tools without cross-system data sharing) and papers addressing medication reconciliation or prescribing errors without a clear link to digital health fragmentation were also excluded.

For studies addressing research question 1 only, research had to be conducted in the United Kingdom and papers published before 2015 were excluded to ensure that the descriptions of digital health fragmentation reflected the more recent landscape in the NHS, when EHRs had become more widely adopted. Given the limited evidence base in the context of AMS and anticipated greater transferability of evidence on impact and solutions, inclusion was not restricted to the United Kingdom for research questions 2 and 3.

### Data Extraction

A data extraction form was developed in Microsoft Excel (V.2510, Microsoft). Extracted information included (1) study characteristics (eg, study design, sample, and methods), (2) information on the exposure of interest (eg, digital health fragmentation conceptual classification and definitions), (3) themes developed from the first research question (eg, the state of digital health fragmentation in the NHS, impacts on patient safety, barriers and facilitators to interoperability, and proposed solutions), (4) themes relating to AMS-specific outcomes (eg, impacts on antimicrobial prescribing practices, stewardship, and patient safety), (5) and themes relating to interventions and solutions (eg, descriptions of tools, strategies, and any evidence of their effectiveness). Data from a random sample of 5 included papers were extracted in duplicate (VS, BB, and SG) to ensure consistency. Data from the remaining papers were extracted by 1 reviewer at a time (VS, BB, or SG).

### Study Risk-of-Bias Assessment

Risk of bias was assessed using the Mixed Methods Appraisal Tool for quantitative, qualitative, and mixed methods studies [16,17]. In addition, studies were assessed for their alignment with the review objectives. Alignment was rated as low when digital fragmentation was not the primary focus of the study, and medium when digital fragmentation was the direct focus, but the transferability of the findings was limited, for example, by factors such as a highly specific clinical context or a multicomponent intervention. Each study was appraised in duplicate and discrepancies resolved by a third reviewer (VS, BB, and SG).

### Synthesis Methods

A narrative synthesis was undertaken by one of the lead authors (VS) using framework analysis to organize and interpret the data. Framework analysis is a case-by-theme approach that entails inductive and deductive processes for analysis [18]. In the case of this review, analysis began with categories derived from the review’s research questions. As the team carried out data extraction, additional categories were added to the framework to reflect the data in the included studies.

## Results

### Overview of Included Studies

After deduplication, 9943 papers were identified from the searched databases. Following screening, 12 papers were deemed eligible for inclusion, and an additional 2 eligible papers were identified through citation searches (Figure 2). Most studies (n=10) addressed research question 1 (extent of digital health fragmentation in the United Kingdom) [8,19-27], 3 studies addressed research question 2 (impact of digital health fragmentation on AMS) [28-30], and 1 study addressed research question 3 (solutions to digital health fragmentation in the context of AMS) [31] (Table 2). In line with the inclusion criteria, all studies under research question 1 were conducted in the United Kingdom. Of the remaining studies, 2 were conducted in Australia [29,30], 1 in the United States [28], and 1 in Portugal [31]. Most studies used qualitative methods (n=9) [8,19,20,22,24,28-31], while fewer studies conducted quantitative analyses or mixed methods (n=5) [21,23,25-27]. Most studies were rated as high quality (n=10; see Multimedia Appendix 3 [8,19-24, 26-30]), while lower-quality ratings were often due to insufficient reporting of methods and analyses. However, several studies addressed digital health fragmentation only indirectly within broader investigations of electronic systems and AMS processes [24,28-31], and 1 study had limited generalizability due to its specialty‑specific focus in gynecological oncology [25].

**Figure 2:**
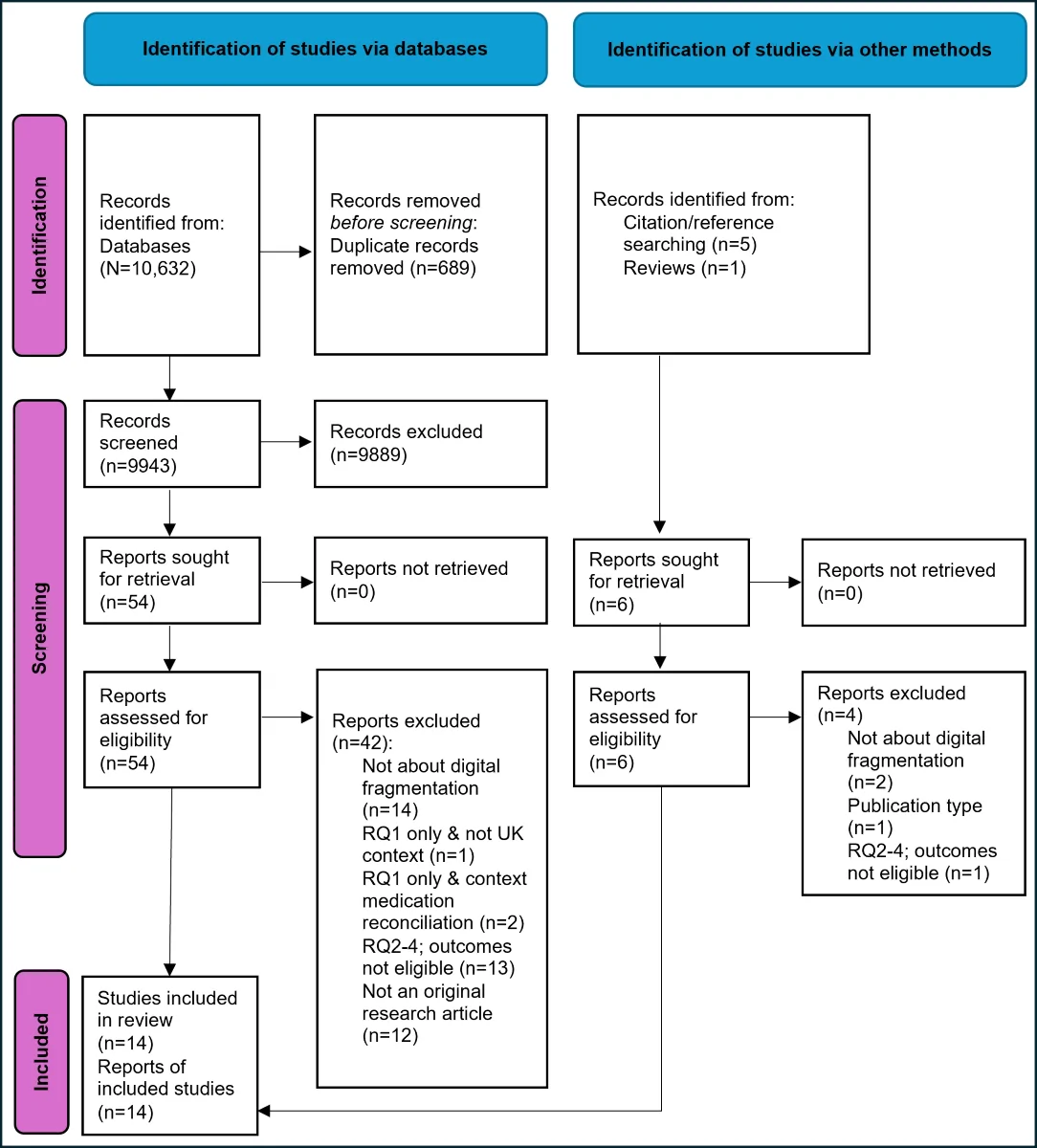
PRISMA (Preferred Reporting Items of Systematic Reviews and Meta-Analyses) flowchart of the literature search and screening. RQ: research question.

**Table 2:** Study characteristics.

First author (year), country			Study’s aim or objective	Study design	Sample
**Research question 1: Digital health fragmentation in the United Kingdom**					
	**Data spread across health care providers or systems**				
		Ahmed et al (2018) [20], England	To explore stakeholder perceptions of the advantages, disadvantages, and patient safety implications of having multiple electronic prescribing systems within a single hospital.	Qualitative interview study	Pharmacists, doctors, and a nurse (n=10) from 4 hospitals
		Clarke et al (2018) [21], England	To identify interorganizational patient-sharing connections in the NHS^a^ in England.	Quantitative/descriptive analysis of administrative data	19,682,360 patients with 130,161,023 secondary care interactions
		Warren et al (2019) [26], England	To identify the frequency of use and spatial distribution of health record systems in the NHS England and to quantify transitions of care between acute hospital trusts and health record systems.	Quantitative descriptive analysis of administrative data	152 NHS England trusts active in 2017-2018; 21,286,873 adults receiving inpatient, outpatient or A&E^b^ care during this period
	**Interoperability, data availability, usability**				
		Cresswell et al (2017) [8], England	To investigate the 2 strategies surrounding integration and interfacing in hospitals that have implemented electronic prescribing systems, to understand the risks to patient safety arising from the failure of information integration and lack of effective information transfer, and to identify potential mitigation approaches.	Qualitative longitudinal interviews, observations, document analysis, and expert roundtables	6 hospitals; 173 interviews with users, implementers, and software suppliers; 24 observations (system use and meetings); 17 implementation document plans; and 2 expert roundtables
		Li et al (2023) [19], England	To capture the perceptions of chief clinical information officers regarding the current state of EHR^c^ interoperability, assess its perceived effect on patient safety, investigate facilitators and barriers to achieving interoperability, and explore perceptions on how the evolution of EHR interoperability would improve patient safety in the coming decade.	Qualitative interview study	15 chief clinical information officers
		Li et al (2024) [22], England	To explore how patients and their caregivers perceive the current status and potential future of EHR interoperability in the English NHS.	Qualitative focus group study	18 patients or non–health care trained caregivers whose care frequently requires visits at 2 or more health care facilities
		Li et al (2025) [23], England	To capture the perceptions of physicians regarding the current state of EHR interoperability, to investigate how a lack of interoperability affects patient care and patient safety, and to estimate the effect of a lack of EHR interoperability on care efficiency and costs.	Quantitative survey study	636 NHS doctors
		Mozaffar et al (2017) [24], England	To review the evidence for roots of reported unintended safety threats associated with the introduction of electronic prescribing in design, implementation, and use, in order to develop a taxonomy of these factors, and use these insights to shed light on possible risk mitigation strategies.	Qualitative longitudinal interviews, observations, and document collection	6 hospitals, 163 interviewees, 24 observations, and 18 documents
		Tookman et al (2025) [25], United Kingdom	To explore multiprofessional perspectives on using EHRs in gynecological oncology and co-design an informatics platform to bring together key information for clinical decision-making in ovarian cancer.	Quantitative survey study and qualitative co-design	92 survey respondents (UK-based professionals working in gynecological oncology); 8 co-design stakeholders
		Zhang et al (2023) [27], England	To characterize the landscape and progression of data-sharing networks in NHS England from 2015 to 2022, and to test the impact of primary to secondary care data-sharing capabilities on clinical quality indicators related to emergency care, patient experience, patient safety, and mortality.	Quantitative descriptive, cross-sectional and longitudinal analysis of administrative data	135 NHS trusts
**Research question 2/3: Impact of digital health fragmentation on AMS^d^ and solutions**					
	Kapadia et al (2018) [28], United States		To understand how AMS programs are structured, implemented, and evolved over time in US hospitals, and to assess what the barriers are in implementing AMS programs.	Qualitative interview study	12 program leaders at 4 hospital-based AMS programs identified as leading in the field of AMS
	Rizvi et al (2025) [30], Australia		To explore the perceived barriers and enablers of community pharmacists’ participation in AMS and the potential strategies to facilitate greater involvement of community pharmacists in AMS initiatives.	Qualitative interview study	20 community pharmacists
	Van Dort et al (2025) [29], Australia		To identify the barriers and facilitators to successful AMS programs in 2 regional hospitals in Australia, and explore the role technology played in supporting AMS.	Qualitative interviews and observations	Observations at 2 tertiary hospitals; interviews with 4 AMS team members
	Simões et al (2018) [31], Portugal		To design and implement a real-time surveillance and clinical decision support system to support AMS implementation, linked with the local AMS strategy, and adapted to the local socio-cultural context.	Qualitative participatory design and interview evaluation	3 hospitals; specific sample sizes not reported

^a^NHS: National Health Service.

^b^A&E: accident and emergency (emergency department).

^c^EHR: electronic health record.

^d^AMS: antimicrobial stewardship.

### Research Question 1: Extent of Digital Health Fragmentation in the United Kingdom

#### Overview

The data were synthesized using framework analysis (Table 3). Ten studies published since 2015 addressed research question 1, which focuses on digital health fragmentation within the UK context. The findings were organized under three themes: (1) digital health fragmentation in the United Kingdom, (2) patient safety risks and care inefficiencies linked to digital health fragmentation, and (3) proposed solutions and future directions (Table 3).

**Table 3:** Themes and subthemes from the framework analysis relating to the review’s research questions.

Research question	Theme	Subthemes
Research question 1: Digital health fragmentation in the UK context	Digital health fragmentation in the United Kingdom	Care fragmentation or distribution of different systems within or across organizational boundaries [8,20,21,23,26] Data-sharing capabilities [27] Perspectives on practical realities of interoperability or usability [19,22,23,25]
	Patient safety risks and care inefficiencies linked to digital health fragmentation	Perceived safety risks due to impaired decision-making and workarounds [8,19,20,22-25] Care inefficiencies and delays [8,19,20,22,23] Quantified impacts on care quality and safety [27]
	Proposed solutions and future directions	Improving system design and usability, for example, through integration or interfacing [8,19,22,24,25] Implementation of common data standards [19] Patient involvement and data comanagement [19,22] Strengthening data security and privacy protections [22] Enhancing digital literacy and workforce training [22] Promoting interorganizational and cross-sector collaboration [19] Organizational strategies [24]
Research question 2: Impact of digital health fragmentation on AMS^a^	Impact of digital health fragmentation on AMS	Fragmented care and digital infrastructure in the context of AMS [28,29,31] Impacts on AMS processes [28-31] Impacts on workflow and clinical interaction [29,31]
Research question 3: Solutions to digital health fragmentation in the AMS context	Solutions to digital health fragmentation in the AMS context	IT solution to integrate data across systems [31]

^a^AMS: antimicrobial stewardship.

#### Digital Health Fragmentation in the United Kingdom

Nine studies described digital health fragmentation, specifically, the landscape of patient and data sharing, and interoperability within the United Kingdom [8,19-23,25-27]. Several studies quantified the extent to which patients receive care across multiple health care providers [21,23,26]. In 2021, 87% (419/484) of surveyed NHS doctors with nonmissing data reported interacting or sharing patients with 3 or more other providers [23]. Clarke et al [21] found that between 2013 and 2015, 26% (4,162,780/16,002,415) of patients presenting multiple times to secondary care within a 12-month period attended more than 1 hospital trust. In 2017-2018, almost 4 million patients had multiple hospital encounters involving different trusts, with 9% (11,017,767/121,351,837) of these encounters occurring at a trust using a different system than previously [26]. With systems from different vendors often not designed to communicate with one another, patient data can become inaccessible. Warren et al [26] identified 21 different EHR vendors and 13 in-house systems across 117 NHS trusts, with 12 operating multiple systems concurrently. Even when nearby hospitals use the same EHR, this only rarely covered 10% or more patient encounters (in just 0.6% of lower layer super output areas) [26].

Zhang et al [27] described the evolution of data-sharing initiatives between NHS primary and secondary care systems since 2015. Between 2015 and 2019, localized solutions were developed to facilitate data sharing between primary care and associated trusts. This sharing was implemented through two models: (1) secure remote access to primary care EHRs via portals, and (2) hospital-controlled data warehouses integrating local primary care data. Coverage of accessible primary care data expanded rapidly from 11.3% of patients in 2015 (n=5,942,682) to nearly 49.5% in 2019 (n=27,090,091) [27]. From 2020 onward, regional integration efforts led to the creation of local care records, supported by common data standards and shared technology vendors. By 2023 (at the time the paper was published), efforts shifted toward national consolidation and full population coverage, aiming for technical capability to share patient data seamlessly between primary and secondary care [27].

Despite technical advancements over the years, multiple studies highlighted challenges in the practical realization of interoperability, including limitations in data sharing and usability from the perspectives of clinicians, patients, and chief clinical information officers [19,22,23,25]. A 2021 survey of NHS doctors across primary and secondary care revealed that 93% (429/461) reported being able to retrieve patients’ medical history via their EHRs, although this access was largely limited to data within their own system. Only 38% (175/460) reported being able to access information from outside their immediate health care setting. Consequently, 96% (396/413) experienced difficulties retrieving information when needed [23]. This was echoed in a survey of clinicians in gynecological oncology (n=92), where most respondents (84/91, 92%) reported accessing multiple EHRs daily, and 60% (54/90) reported lacking comprehensive data to support clinical decision-making [25]. Furthermore, 17% (16/92) stated that searching for information consumed more than half of their clinical time [25]. In interviews (n=15) held in 2020/2021, chief clinical information officers reported that interoperability was often not considered during procurement of new EHRs, resulting in restricted data sharing [19]. This led to fragmentation of patient records across multiple sources, poor data quality, and workflow inefficiencies [19].

Beyond cross-organizational fragmentation, 2 studies explored interoperability and usability issues occurring within organizational boundaries [8,20]. Hospitals operating multiple electronic prescribing systems reported challenges, including duplication of tasks, workflow disruptions, training gaps, increased workload, and technical issues requiring workarounds [20]. Cresswell et al [8] noted that while hospital-wide multimodular integrated platforms (see Table 1 for used terms and definitions) reduced some risks and usability issues, they were costly and inflexible, whereas stand-alone systems (ie, not integrated into 1 platform) created ongoing safety and usability concerns.

#### Patient Safety Risks and Care Inefficiencies Linked to Digital Health Fragmentation

Eight studies examined the implications of digital health fragmentation for patient safety and care efficiency. Digital health fragmentation was frequently reported to undermine clinical decision-making, primarily due to data quality issues, such as incomplete, inaccurate, or incoherent information; delays in accessing results; and the need for manual workarounds [8,19,20,22-25]. Consequences included an incomplete clinical picture (eg, missing medication histories or life-threatening allergy information) [19,20], suboptimal care planning and coordination [19], increased workloads [8], delayed diagnosis [20] and care [8], and placing responsibility on patients to recall or verify their clinical history [22]. Duplication of tests and investigations, particularly infection panels, radiology, blood work, and urine analyses, was also frequently reported [19,22,23]. For example, in focus groups held in 2022, patients with chronic conditions or polypharmacy reported having to recall medication histories, undergo repeated investigations, and experience inaccurate or incomplete documentation between primary and secondary care, resulting in inconvenience, disrupted continuity of care, and inefficient use of clinical time [19].

NHS doctors across primary and secondary care reported difficulties accessing timely information, which negatively impacted clinical workflows, consultation time, information sharing among professionals, and patient safety [23]. Perceptions of safety risks linked to limited interoperability were especially pronounced in pediatrics, psychiatry, surgery, and accident and emergency where 62%-69% of surveyed doctors identified this as a concern (compared with 29%-48% of doctors with other specialty training, including internal medicine, anesthesia, and general practice) [23].

Beyond safety, limited interoperability was also linked to longer consultations and extended hospital stays [23]. Analyzing administrative data, Zhang et al [27] found that the ability to share data between primary and secondary care was significantly associated with reduced breaches of the accident and emergency 4-hour discharge target and improved patient experience in emergency care [27].

#### Proposed Solutions and Future Directions

Five qualitative studies explored proposed solutions for improving EHR as well as electronic prescribing system interoperability [8,19,22,24,25]. All reported the need to improve system design for better integration of information and usability by having more user-centric, intuitive, and flexible design of interfaces that align with the clinical workflow [8,19,22,24,25]. Innovative approaches, such as leveraging artificial intelligence to automate data retrieval, were suggested as ways to reduce manual tasks and improve information quality [24]. Organizational strategies were also deemed essential, including short- and long-term plans for optimization of electronic prescribing systems [24]. Cresswell et al [8] reported that sites using stand-alone electronic prescribing systems developed effective interfacing solutions to address interoperability gaps.

Patients expressed a strong preference for a single, centralized NHS system to facilitate easy access and data sharing [22]. Several studies underscored the importance of patient involvement in comanaging their records, with or without write access, to ensure data accuracy and reduce clinician workload [19,22]. Other recommendations included implementing common data standards [19], strengthening data security and privacy protections [22], improving staff digital literacy and workforce training [22], and promoting interorganizational and cross-sector collaboration (including allied health services and social care) [19].

Tookman et al [25] described the co-design of an integrated informatics platform for gynecological oncology, using algorithms and natural language processing to collate information from disparate systems into a single interface. This was co-designed by Imperial College Healthcare NHS Trust’s gynecological oncology team. Further development and evaluation of this platform are planned [25].

### Research Question 2: Impact of Digital Health Fragmentation on AMS

Only indirect evidence from 4 studies was found in relation to impacts of digital health fragmentation on AMS, with evidence from the United States, Australia, and Portugal [28-31]. These studies highlighted that effective AMS requires the use of multiple systems, such as microbiology software, EHRs, pharmacy, and AMS software [28,29]. Health care professionals reported that poor interoperability between these systems posed a significant barrier to stewardship activities [28,29,31]. Time spent retrieving patient histories disrupted clinical workflows and reduced opportunities for direct patient interaction [29,31]. Limited interoperability and inadequate data sharing were perceived to negatively affect communication among health care professionals [29] and between general practice and community pharmacy [30] in the context of AMS. In Australia, limited access to shared records was seen as contributing to antimicrobial misuse and impeding AMS strategies, such as delayed antibiotic prescribing [30].

### Research Question 3: Solutions to Digital Health Fragmentation in AMS

This review identified only 1 study evaluating a specific intervention aimed at reducing fragmentation for AMS. Simões et al [31] described the development of HAITooL (a tool kit to prevent, manage, and control health care–associated infections in Portugal). HAITooL is a clinical decision support system designed to support AMS by integrating data from multiple hospital systems, presenting a unified timeline with microbiology results and clinical history, and providing real-time alerts and surveillance data. The data integration functionality aims to mitigate issues arising from fragmented data and poor interoperability across 3 different hospitals and regions within the Portuguese health care context [31]. Interviewed health care professionals valued the tool for enhancing communication, situational awareness, and AMS efficiency [31]. However, the evaluation did not include objective data, and the effects of data integration could not be separated from effects of other features of the system.

## Discussion

### Principal Results

This review aimed to (1) characterize the extent of digital health fragmentation in the United Kingdom, (2) summarize the evidence on its impacts on AMS, and (3) identify any relevant solutions. The findings indicate that digital health fragmentation remains widespread within the United Kingdom and likely affects a large proportion of patients, with an estimated 26% (over 4 million) of patients with multiple hospital encounters within a year moving between different health care organizations [21]. Although national and regional initiatives have improved data-sharing capabilities, data availability, interoperability, and usability challenges persist, impacting the availability of clinical information in everyday workflows.

Digital health fragmentation was found to compromise patient safety, patient experience, and care efficiency. Included studies reported impacts such as delays in accessing patient data, high clinical workloads, manual workarounds, suboptimal care coordination, duplication of tests, delayed diagnoses and treatment, extended consultations, accident and emergency waits, and longer hospital stays. These findings are similar to those of previous reviews. Although no previous review has specifically examined digital health fragmentation in the context of AMS, several reviews have explored interoperability, data sharing, and patient safety more broadly. Given the well-documented risks care fragmentation poses to patient safety [32], previous reviews have focused on the role of IT and interoperability in mitigating patient harm and have identified interoperability and system usability as key facilitators and barriers to medication and patient safety [10,33-36]. While these reviews do not address AMS directly, their findings are highly relevant to antimicrobial prescribing, which similarly depends on timely access to accurate information, such as prior microbiology or prescribing history, to support appropriate treatment decisions. Yet, our review identified very few studies that addressed the relationship between digital health fragmentation and AMS. Only 4 studies provided relevant, largely indirect insights, and only 1 study reported a specific intervention designed to mitigate fragmentation for AMS purposes.

### Strengths and Limitations of the Review and Included Evidence

This review’s strengths include its novelty in addressing digital health fragmentation with a specific focus on AMS, an important and underexamined topic. Although the identified evidence base was limited, a limitation of the review, this is an important finding in itself, as it highlights a gap in the literature and existing knowledge explicitly examining how digital health fragmentation affects antimicrobial prescribing, AMS effectiveness, the downstream implications for patient outcomes and resistance, and potential solutions.

We used an inclusive conceptualization of digital health fragmentation to encompass data spread, data-sharing capabilities, interoperability, and usability. We believe these factors must be considered together. For instance, interoperability can be defined in multiple ways, and our findings suggest that the effectiveness of data sharing and interoperability is dependent on system usability within the clinical workflow. Furthermore, we included both qualitative and quantitative research to capture a broader picture of the evidence. Although only a few studies were identified that quantified the extent or impact of digital health fragmentation, qualitative studies provided valuable insights into users’ day-to-day experiences. A further strength of this review is that its first research question examines digital health fragmentation within the context of the UK health system specifically. While understood as a centrally organized national service, the NHS functions practically as a federation of semiautonomous organizations. This structure has implications for interoperability as different procurement decisions are made locally, making the review’s findings relevant to other decentralized health systems.

Beyond the limited evidence base, limitations of the review methodology need to be acknowledged. There remains a possibility that relevant research was missed due to the rapid search strategy (limited number and choice of databases, search terms, and limits) and screening process (partial dual screening). For example, literature relevant to health informatics, implementation science, and digital health may be indexed on databases not included in the searches. However, these streamlined choices reflect methods commonly used to balance rigor with timeliness in rapid reviews [11,37]. Furthermore, we screened a large number of papers and supplemented this with reference screening of relevant reviews, and citation tracking and reference checks of the included studies to minimize bias.

### Implications

Digital health fragmentation within the AMS context remains underresearched and the conduct of a full systematic review on this topic is not warranted, given the current evidence base retrieved in this rapid review. Targeted studies are needed to understand the extent and consequences of digital health fragmentation and to generate evidence‑based recommendations that maximize the benefits of electronic systems while minimizing the risks associated with incomplete or siloed patient data. There is also a need for more qualitative evidence involving patients on how digital health fragmentation impacts their lived experience in the context of antimicrobial prescribing.

Qualitative evidence from clinicians suggests that even when data are theoretically available, practical usability issues often prevent clinicians from accessing or applying the information they need at the point of care. Future research should therefore distinguish clearly between data availability and data usability, recognizing that both technical interoperability and real‑world workflow integration are essential for effective clinical care, likely including AMS.

Such future research efforts would also have wider implications. With the rapidly evolving health technology landscape, the use of multiple digital systems and the challenge of integrating them are increasingly unavoidable. AMS is a well-recognized global priority and presents an opportunity to drive solutions for addressing digital health fragmentation more broadly. If systems can successfully interoperate in this domain, solutions can likely yield benefits for many other areas of clinical care. Moreover, the implications of solutions to digital health fragmentation extend beyond individual antimicrobial prescribing decisions. Linked, high‑quality data are also critical for AMS evaluation and AMR epidemiology: without integrated records, it is difficult to monitor antimicrobial use across organizations, identify whether local prescribing practices contribute to emerging resistance patterns, or determine which stewardship interventions are most effective. Furthermore, fragmented data environments are also relevant to the adoption of artificial intelligence–driven clinical decision support tools. Models trained or deployed on incomplete or poorly linked data risk embedding biases and generating unsafe recommendations.

In the United Kingdom, national research and development programs and the emerging Secure Data Environment landscape [38] are currently oriented primarily toward supporting data sharing for research rather than for direct care or population health. At present, the OneLondon Secure Data Environment [39] is the only example of a joint governance model that brings together direct care, population health, and research uses under a unified framework. Without similar integrated governance approaches more widely, there is a risk that data remain siloed, not used to maximize value, duplicate efforts, and poorly understood by the public.

While commonly described as a technical problem, interoperability is also a social and political one within the NHS context. The NHS operates as a federation of independent trusts and general practitioner practices, each procuring and managing their own systems. Additionally, there is also substantial digital fragmentation within social care and lack of data sharing between health and social care. As a result, data are generated and governed across organizational boundaries that do not easily align. In a fully centralized system with unified governance, interoperability would be simpler. Thus, solutions also need to consider governance, incentives, and organizational arrangements alongside technical standards.

### Conclusions

Safe antimicrobial prescribing depends on access to comprehensive microbiology, resistance, and prescribing histories. Digital health fragmentation in the NHS affects a substantial number of patients who receive care from multiple health care organizations using different electronic systems, resulting in fragmented patient data across platforms. Yet, while the broader literature demonstrates clear implications of digital health fragmentation for patient safety and efficiency, evidence directly addressing digital health fragmentation in antimicrobial prescribing and AMS remains limited, based on largely indirect evidence from other high-income countries. Further research is needed to quantify the scale and consequences of digital health fragmentation in AMS and to evaluate the potential of solutions to mitigate these risks.

## Data Availability

All data generated or analyzed during this study are included in this published paper.

## Appendix

Multimedia Appendix 1 PRISMA (Preferred Reporting Items of Systematic Reviews and Meta-Analyses) checklist.

Multimedia Appendix 2 Search strategy.

Multimedia Appendix 3 Quality appraisal.

## Abbreviations

AMR: antimicrobial resistance
AMS: antimicrobial stewardship
EHR: electronic health record
NHS: National Health Service
PRISMA: Preferred Reporting Items of Systematic Reviews and Meta-Analyses

## References

[1] Warren L, Clarke J, Darzi A (2020). Measuring the scale of hospital health record system fragmentation in England. Health Serv Res.

[2] Baltas I, Stockdale T, Tausan M, Kashif A, Anwar J, Anvar J, Koutoumanou E, Sidebottom D, Garcia-Arias V, Wright M, Democratis J (2021). Impact of antibiotic timing on mortality from gram-negative bacteraemia in an English district general hospital: the importance of getting it right every time. J Antimicrob Chemother.

[3] Bauer KA, Kullar R, Gilchrist M, File TM (2019). Antibiotics and adverse events: the role of antimicrobial stewardship programs in 'doing no harm'. Curr Opin Infect Dis.

[4] Rawson TM, Wilson RC, O'Hare D, Herrero P, Kambugu A, Lamorde M, Ellington M, Georgiou P, Cass A, Hope WW, Holmes AH (2021). Optimizing antimicrobial use: challenges, advances and opportunities. Nat Rev Microbiol.

[5] (2026). Antibiotic use: target ≥70% of total antibiotic use being access group antibiotics (70% Access target ). World Health Organization.

[6] (2023). English surveillance programme for antimicrobial utilisation and resistance (ESPAUR) report 2022 to 2023 UKHSA. UK Health Security Agency (UKHSA).

[7] Antimicrobial Resistance Collaborators (2022). Global burden of bacterial antimicrobial resistance in 2019: a systematic analysis. Lancet.

[8] Cresswell KM, Mozaffar H, Lee L, Williams R, Sheikh A (2017). Safety risks associated with the lack of integration and interfacing of hospital health information technologies: a qualitative study of hospital electronic prescribing systems in England. BMJ Qual Saf.

[9] (2023). Interoperability. NHS England.

[10] Li E, Clarke J, Ashrafian H, Darzi A, Neves AL (2022). The impact of electronic health record interoperability on safety and quality of care in high-income countries: systematic review. J Med Internet Res.

[11] Tricco AC, Langlois E, Straus SE (2017). Rapid Reviews to Strengthen Health Policy and Systems: A Practical Guide.

[12] Stevens A, Hersi M, Garritty C, Hartling L, Shea BJ, Stewart LA, Welch VA, Tricco AC, Cochrane Rapid Reviews Methods Group (2025). Rapid review method series: interim guidance for the reporting of rapid reviews. BMJ Evid Based Med.

[13] Page MJ, McKenzie JE, Bossuyt PM, Boutron I, Hoffmann TC, Mulrow CD, Shamseer L, Tetzlaff JM, Akl EA, Brennan SE, Chou R, Glanville J, Grimshaw JM, Hróbjartsson A, Lalu MM, Li T, Loder EW, Mayo-Wilson E, McDonald S, McGuinness LA, Stewart LA, Thomas J, Tricco AC, Welch VA, Whiting P, Moher D (2021). The PRISMA 2020 statement: an updated guideline for reporting systematic reviews. BMJ.

[14] Ouzzani M, Hammady H, Fedorowicz Z, Elmagarmid A (2016). Rayyan-a web and mobile app for systematic reviews. Syst Rev.

[15] Higgins J, Thomas J, Chandler J (2024). Cochrane handbook for systematic reviews of interventions version 6.5. Cochrane.

[16] Pace R, Pluye P, Bartlett G, Macaulay AC, Salsberg J, Jagosh J, Seller R (2012). Testing the reliability and efficiency of the pilot Mixed Methods Appraisal Tool (MMAT) for systematic mixed studies review. Int J Nurs Stud.

[17] Pluye P, Hong QN (2014). Combining the power of stories and the power of numbers: mixed methods research and mixed studies reviews. Annu Rev Public Health.

[18] Gale NK, Heath G, Cameron E, Rashid S, Redwood S (2013). Using the framework method for the analysis of qualitative data in multi-disciplinary health research. BMC Med Res Methodol.

[19] Li E, Lounsbury O, Clarke J, Ashrafian H, Darzi A, Neves AL (2023). Perceptions of chief clinical information officers on the state of electronic health records systems interoperability in NHS England: a qualitative interview study. BMC Med Inform Decis Mak.

[20] Ahmed Z, Jani Y, Franklin BD (2018). Qualitative study exploring the phenomenon of multiple electronic prescribing systems within single hospital organisations. BMC Health Serv Res.

[21] Clarke JM, Warren LR, Arora S, Barahona M, Darzi AW (2018). Guiding interoperable electronic health records through patient-sharing networks. NPJ Digit Med.

[22] Li E, Lounsbury O, Clarke J, Ashrafian H, Darzi A, Neves AL (2024). Patient and caregiver perceptions of electronic health records interoperability in the NHS and its impact on care quality: a focus group study. BMC Med Inform Decis Mak.

[23] Li E, Lounsbury O, Hasnain M, Ashrafian H, Darzi A, Neves AL, Clarke J (2025). Physician experiences of electronic health record interoperability and its practical impact on care delivery in the English NHS: a cross-sectional survey study. BMJ Open.

[24] Mozaffar H, Cresswell KM, Williams R, Bates DW, Sheikh A (2017). Exploring the roots of unintended safety threats associated with the introduction of hospital ePrescribing systems and candidate avoidance and/or mitigation strategies: a qualitative study. BMJ Qual Saf.

[25] Tookman L, Lear R, Abdullahi YS, Samani A, Averill P, Hunt A, Papadimitriou D, Nkolobe BE, Ghaem-Maghami S, Glampson B, A McNeish I, Mayer EK (2025). Understanding and addressing challenges with electronic health record use in gynecological oncology: cross-sectional survey of multidisciplinary professionals in the United Kingdom and co-design of an integrated informatics platform to support clinical decision-making. JMIR Cancer.

[26] Warren LR, Clarke J, Arora S, Darzi A (2019). Improving data sharing between acute hospitals in England: an overview of health record system distribution and retrospective observational analysis of inter-hospital transitions of care. BMJ Open.

[27] Zhang J, Ashrafian H, Delaney B, Darzi A (2023). Impact of primary to secondary care data sharing on care quality in NHS England hospitals. NPJ Digit Med.

[28] Kapadia SN, Abramson EL, Carter EJ, Loo AS, Kaushal R, Calfee DP, Simon MS (2018). The expanding role of antimicrobial stewardship programs in hospitals in the United States: lessons learned from a multisite qualitative study. Jt Comm J Qual Patient Saf.

[29] Van Dort BA, Carland JE, Penm J, Ritchie A, Morton K, Chahoud N, Baysari MT (2025). Antimicrobial stewardship in regional hospitals: a human factors evaluation of barriers and facilitators and the role of technology. Intern Med J.

[30] Rizvi T, Zaidi STR, Williams M, Thompson A, Peterson GM (2025). Factors influencing community pharmacists' participation in antimicrobial stewardship: a qualitative inquiry. Pharmacy (Basel).

[31] Simões AS, Maia M, Gregório J, Couto I, Asfeldt A, Simonsen G, Póvoa P, Viveiros M, Lapão L V (2018). Participatory implementation of an antibiotic stewardship programme supported by an innovative surveillance and clinical decision-support system. J Hosp Infect.

[32] Snow K, Galaviz K, Turbow S (2020). Patient outcomes following interhospital care fragmentation: a systematic review. J Gen Intern Med.

[33] Dobrow MJ, Bytautas JP, Tharmalingam S, Hagens S (2019). Interoperable electronic health records and health information exchanges: systematic review. JMIR Med Inform.

[34] Hyvämäki P, Kääriäinen M, Tuomikoski A, Pikkarainen M, Jansson M (2022). Registered nurses' and medical doctors' experiences of patient safety in health information exchange during interorganizational care transitions: a qualitative review. J Patient Saf.

[35] Mondal R, Sameer M (2025). Connected healthcare system technology interventions to improve patient safety by reducing medical errors: a systematic review. Glob J Qual Saf Healthc.

[36] Cahill M, Cleary BJ, Cullinan S (2025). The influence of electronic health record design on usability and medication safety: systematic review. BMC Health Serv Res.

[37] Langlois EV, Straus SE, Antony J, King VJ, Tricco AC (2019). Using rapid reviews to strengthen health policy and systems and progress towards universal health coverage. BMJ Glob Health.

[38] Secure data environment. NHS England Digital.

[39] The London secure data environment. OneLondon.

[40] (2026). Copilot. Microsoft.

[41] (2025). Career development award. UK Research and Innovation.

