# Supplementary material for: Extent of Digital Health Fragmentation and Potential Implications for Antimicrobial Prescribing: Rapid Evidence Review
Source: J Med Internet Res. 2026 Oct 6;28:e94014. doi: 10.2196/94014 (PMC13684119; doi:10.2196/94014)
Supplement: Supplementary file 2 [file jmir_v28i1e94014_app2.docx]

## Multimedia Appendix 2

## Search strategy

**MEDLINE ALL**

| 1 | exp Health Information Exchange/ | 1,200 |
| --- | --- | --- |
| 2 | exp Medical Record Linkage/ | 4,874 |
| 3 | exp Health Information Interoperability/ | 384 |
| 4 | exp Systems Integration/ | 9,705 |
| 5 | ((digital or system* or electronic or record* or data or information) adj2 (fragment* or connect* or coordinat* or integrat* or shar* or exchang* or interoperab* or interfac* or transfer*)).mp. | 180,567 |
| 6 | 1 or 2 or 3 or 4 or 5 | 184,277 |
| 7 | exp Antimicrobial Stewardship/ | 4,288 |
| 8 | exp Drug Utilization Review/ | 8,134 |
| 9 | exp Drug Resistance, Microbial/ | 195,745 |
| 10 | exp Patient Safety/ | 27,757 |
| 11 | exp Risk Management/ | 373,950 |
| 12 | exp Medication Errors/ | 21,472 |
| 13 | exp Inappropriate Prescribing/ | 5,203 |
| 14 | ("antimicrobial prescri*" or "antibiotic prescri*" or "antimicrobial use" or "antibiotic use" or "antimicrobial stewardship" or "stewardship program*" or "antimicrobial resistance" or "antibiotic resistance").mp. | 132,604 |
| 15 | ((patient adj2 safety) or (patient adj2 incident*) or (patient adj2 outcome*) or (patient adj2 harm) or "medic* error*" or "drug error*" or "drug toxicity" or "drug prescri*" or "medic* prescri*" or "prescri* practices" or "prescri* behav*" or "prescrib* pattern*" or (safe* adj1 prescri*) or "prescri* error*").mp. | 341,306 |
| 16 | 7 or 8 or 9 or 10 or 11 or 12 or 13 or 14 or 15 | 970,157 |
| 17 | exp United Kingdom/ | 406,627 |
| 18 | exp England/ | 117,512 |
| 19 | ("United Kingdom" or "UK" or England).mp. | 500,701 |
| 20 | 17 or 18 or 19 | 547,942 |
| 21 | (health* or patient* or clinic* or hospital or medic* or drug* or "primary care" or "secondary care" or "general practi*" or "GP").mp. | 20,329,875 |
| 22 | 20 and 21 | 403,760 |
| 23 | ("National Health Service" or "NHS").mp. | 56,535 |
| 24 | 22 or 23 | 426,638 |
| 25 | 6 and 24 | 3,794 |
| 26 | 6 and 16 | 9,106 |
| 27 | 25 or 26 | 12,487 |
| 28 | Limit 27 to English language | 12,196 |
| 29 | Limit 28 to yr=”2010-Current” | **9,538** |

**PsycInfo**

| 1 | ((digital or system* or electronic or record* or data or information) adj2 (fragment* or connect* or coordinat* or integrat* or shar* or exchang* or interoperab* or interfac* or transfer*)).mp. | 45,570 |
| --- | --- | --- |
| 2 | exp Antibiotics/ | 3,129 |
| 3 | exp Drug Resistance/ | 253 |
| 4 | exp Patient Safety/ | 4,721 |
| 5 | exp Risk Management/ | 118,881 |
| 6 | exp "Prescribing (Drugs)"/ | 4,798 |
| 7 | ("antimicrobial prescri*" or "antibiotic prescri*" or "antimicrobial use" or "antibiotic use" or "antimicrobial stewardship" or "stewardship program*" or "antimicrobial resistance" or "antibiotic resistance").mp. | 960 |
| 8 | ((patient adj2 safety) or (patient adj2 incident*) or (patient adj2 outcome*) or (patient adj2 harm) or "medic* error*" or "drug error*" or "drug toxicity" or "drug prescri*" or "medic* prescri*" or "prescri* practices" or "prescri* behav*" or "prescrib* pattern*" or (safe* adj1 prescri*) or "prescri* error*").mp. | 35,574 |
| 9 | 2 or 3 or 4 or 5 or 6 or 7 or 8 | 52,971 |
| 10 | ("National Health Service" or "NHS").mp. | 8,317 |
| 11 | ("United Kingdom" or "UK" or England).mp. | 95,446 |
| 12 | (health* or patient* or clinic* or hospital or medic* or drug* or "primary care" or "secondary care" or "general practi*" or "GP").mp. | 2,381,731 |
| 13 | (11 and 12) or 10 | 57,633 |
| 14 | 1 and 13 | 557 |
| 15 | 1 and 9 | 829 |
| 16 | 14 or 15 | 1,355 |
| 17 | Limit 16 to English language | 1,327 |
| 18 | Limit 17 to yr=”2010-Current” | **1,094** |

**Cochrane Reviews**

Note that the search on this database was limited to the list of terms relating to digital health fragmentation only to be more inclusive.

| 1 | MeSH descriptor: [Health Information Exchange] explode all trees | |
| --- | --- | --- |
| 2 | MeSH descriptor: [Medical Record Linkage] explode all trees | |
| 3 | MeSH descriptor: [Health Information Interoperability] explode all trees | |
| 4 | MeSH descriptor: [Systems Integration] explode all trees | |
| 5 | ((digital or system* or electronic or record* or data or information) NEAR/1 (fragment* or connect* or coordinat* or integrat* or shar* or exchang* or interoperab* or interfac* or transfer*)):ti,ab,kw  (Word variations have been searched) | |
| 6 | #1 or #2 or #3 or #4 or #5 | **39** |
