# Supplementary material for: Extent of Digital Health Fragmentation and Potential Implications for Antimicrobial Prescribing: Rapid Evidence Review
Source: J Med Internet Res. 2026 Oct 6;28:e94014. doi: 10.2196/94014 (PMC13684119; doi:10.2196/94014)
Supplement: Supplementary file 3 [file jmir_v28i1e94014_app3.docx]

## Multimedia Appendix 3

## Quality appraisal

Multimedia Appendix Table 1. Quality appraisal of included studies

| MMAT categories | Ahmed et al (2018) [20] | Clarke et al (2018) [21] | Cresswell et al (2017) [8] | Li et al (2023) [19] | Li et al (2024) [22] | Li (2025) [23] | Mozaffar et al (2017) [24] | Tookman et al (2025) [25] | Warren et al (2019) [26] | Zhang et al (2023) [27] | Kapadia et al (2018) [28] | Rizvi et al (2025) [30] | Van Dort et al (2025) [29] | Simões et al (2018) [31] |
| --- | --- | --- | --- | --- | --- | --- | --- | --- | --- | --- | --- | --- | --- | --- |
| S1. Are there clear RQs? | Y | Y | Y | Y | Y | Y | Y | Y | Y | Y | Y | Y | Y | Y |
| S2. Do collected data allow to address RQs? | Y | Y | Y | Y | Y | Y | Y | Y | Y | Y | Y | Y | Y | Y |
| 1. Qualitative | | | | | | | | | | | | | | |
| 1.1. Is the qualitative approach appropriate to answer the RQ? | Y |  | Y | Y | Y |  | Y | Y |  |  | Y | Y | Y | Y |
| 1.2. Are the qualitative data collection methods adequate to address the RQ? | Y |  | Y | Y | Y |  | Y | Y |  |  | Y | Y | Y | Y |
| 1.3. Are the findings adequately derived from the data? | Y |  | Y | Y | Y |  | Y | N |  |  | Y | Y | Y | CT |
| 1.4. Is the interpretation of results sufficiently substantiated by data? | Y |  | Y | Y | Y |  | Y | CT |  |  | CT | Y | Y | CT |
| 1.5. Is there coherence between qualitative data sources, collection, analysis and interpretation? | Y |  | Y | Y | Y |  | Y | CT |  |  | Y | Y | Y | CT |
| 2. Quantitative randomised controlled trials | | | | | | | | | | | | | | |
| 2.1. Is randomisation appropriately performed? |  |  |  |  |  |  |  |  |  |  |  |  |  |  |
| 2.2. Are the groups comparable at baseline? |  |  |  |  |  |  |  |  |  |  |  |  |  |  |
| 2.3. Are there complete outcome data? |  |  |  |  |  |  |  |  |  |  |  |  |  |  |
| 2.4. Are outcome assessors blinded to the intervention provided? |  |  |  |  |  |  |  |  |  |  |  |  |  |  |
| 2.5 Did the participants adhere to the assigned intervention? |  |  |  |  |  |  |  |  |  |  |  |  |  |  |
| 3. Quantitative nonrandomised | | | | | | | | | | | | | | |
| 3.1. Are the participants representative of the target population? |  |  |  |  |  |  |  |  |  |  |  |  |  |  |
| 3.2. Are measurements appropriate regarding both the outcome and intervention (or exposure)? |  |  |  |  |  |  |  |  |  |  |  |  |  |  |
| 3.3. Are there complete outcome data? |  |  |  |  |  |  |  |  |  |  |  |  |  |  |
| 3.4. Are the confounders accounted for in the design and analysis? |  |  |  |  |  |  |  |  |  |  |  |  |  |  |
| 3.5. During the study period, is the intervention administered (or exposure occurred) as intended? |  |  |  |  |  |  |  |  |  |  |  |  |  |  |
| 4. Quantitative descriptive | | | | | | | | | | | | | | |
| 4.1. Is the sampling strategy relevant to address the RQ? |  | Y |  |  |  | Y |  | Y | Y | Y |  |  |  | Y |
| 4.2. Is the sample representative of the target population? |  | Y |  |  |  | Y |  | CT | Y | Y |  |  |  | CT |
| 4.3. Are the measurements appropriate? |  | Y |  |  |  | Y |  | Y | Y | Y |  |  |  | Y |
| 4.4. Is the risk of nonresponse bias low? |  | Y |  |  |  | CT |  | N | Y | Y |  |  |  | CT |
| 4.5. Is the statistical analysis appropriate to answer the RQ? |  | Y |  |  |  | Y |  | Y | Y | Y |  |  |  | CT |
| 5. Mixed methods | | | | | | | | | | | | | | |
| 5.1. Is there an adequate rationale for using a mixed methods design to address the RQ? |  |  |  |  |  |  |  |  |  |  |  |  |  |  |
| 5.2. Are the different components of the study effectively integrated to answer the RQ? |  |  |  |  |  |  |  |  |  |  |  |  |  |  |
| 5.3. Are the outputs of the integration of qualitative and quantitative components adequately interpreted? |  |  |  |  |  |  |  |  |  |  |  |  |  |  |
| 5.4. Are divergences and inconsistencies between quantitative and qualitative results adequately addressed? |  |  |  |  |  |  |  |  |  |  |  |  |  |  |
| 5.5. Do the different components of the study adhere to the quality criteria of each tradition of the methods involved? |  |  |  |  |  |  |  |  |  |  |  |  |  |  |
| **Alignment with review’s RQ** | H | H | H | H | H | H | L | M | H | H | L | L | L | M |

**Notes**: Most studies were appraised using the five MMAT questions relevant to their specific study design. For studies that employed multiple methods across different research questions but did not qualify as mixed-methods studies, the appropriate MMAT sections were applied to each research question. **Abbreviations**: CT = can’t tell; H = high; L = low; M = medium; MMAT = Mixed Methods Appraisal Tool; RQ = research question; N = no; RQ = research question; Y = yes
